# Physical performance in patients treated with nocturnal hemodialysis - a systematic review of the evidence

**DOI:** 10.1186/s12882-019-1518-4

**Published:** 2019-08-14

**Authors:** Manouk Dam, Peter J. M. Weijs, Frans J. van Ittersum, Brigit C. van Jaarsveld

**Affiliations:** 1 Department of Nutrition and Dietetics, Amsterdam University Medical Centers, VU University, Amsterdam, the Netherlands; 2 Department of Nephrology, Amsterdam University Medical Centers, VU University, Amsterdam, the Netherlands

**Keywords:** Chronic hemodialysis, Dialysis, Exercise, ESRD, Physical activity

## Abstract

**Background:**

Patients treated with conventional hemodialysis have poor physical performance, explained by insufficient metabolic clearance and shortage of time by time-consuming dialysis. Nocturnal hemodialysis improves metabolic control and results in increased spare time. Our aim is to investigate whether physical performance in nocturnal hemodialysis is superior to conventional hemodialysis.

**Methods:**

A systematic search was conducted in MEDLINE, Embase, CINAHL, PhycInfo and Web of Science until January 2018. Primary outcomes were physical performance, activity, strength and muscle mass in home or in-center nocturnal hemodialysis. Methodological quality was assessed with the Newcastle-Ottawa scale.

**Results:**

Ten studies met the inclusion criteria, including 2 RCTs, evaluating 526 nocturnal hemodialysis patients with a mean follow-up of 15, 3 months. The methodological quality of 4 studies was limited. Physical capacity tests were done in 3 studies with different methodology: short-physical performance battery, exercise spirometry and 6-min walk test. The latter 2 showed significant improvements in physical performance. Four studies assessed lean mass using dual-energy X-ray absorptiometry (2×) and bioelectrical impedance analysis (2×), of which 1 demonstrated increased lean body and skeletal muscle mass. In 5 studies a Quality of Life questionnaire was used, of which 2 showed improved physical component score.

**Conclusions:**

The evidence on the effect of nocturnal hemodialysis on physical performance is either of insufficient methodological quality or only measures isolated aspects of physical performance. As literature emphasizes the importance of physical activity on clinical outcomes, it is necessary to conduct larger studies of high methodological quality using capacity tests for answering the question whether nocturnal hemodialysis can improve physical performance of patients with end-stage renal disease.

**Trial registration:**

NTR4715, Netherlands Trial Register. Registered 30 July 2014.

**Electronic supplementary material:**

The online version of this article (10.1186/s12882-019-1518-4) contains supplementary material, which is available to authorized users.

## Background

Hemodialysis (HD) patients are known to have poor physical performance in comparison with healthy subjects [1–3]. Insufficient physical performance is associated with poor clinical outcomes, such as quality of life (QoL) and overall health and survival [2, 4–7]. During the last years this issue has drawn more attention, although the National Kidney Foundation already emphasized in 2005 the importance of frequent exercise to benefit cardiovascular health and other clinical outcomes. Whether physical performance improves when patients switch from conventional hemodialysis (CHD) to a nocturnal hemodialysis (NHD) regimen is unclear.

Low activity levels in HD patients have a multifactorial cause [8]. First, a state of chronic fatigue occurs during dialysis, probably because the metabolic clearance that can be offered through peritoneal or hemodialysis is largely insufficient in comparison with normal kidney function. Secondly, patients often suffer from multiple comorbidities, leading to a lower general activity pattern i.e. a more passive lifestyle. Also, protein-energy wasting is often present in patients with end-stage renal disease (ESRD), defined as an inflammatory state leading to diminished muscle mass and strength [9, 10], which can have a major impact on the ability to exercise [11]. At last, we should not forget the time-burden patients perceive by a time-consuming dialysis schedule, preventing them from exercising on a regular basis.

The beneficial impact on clinical parameters in dialysis patients, when they increase their daily activity pattern, has been well established. Heiwe et al. [12] reviewed the effect of exercise training on different health outcomes in 41 trials and found improvements in anaerobic capacity, muscle- and cardiovascular functioning and QoL. Smart et al. [13] showed in a meta-analysis improvements in VO_2peak_ and lean body mass when dialysis patients followed an exercise program.

NHD, characterized by long dialysis sessions during nighttime, improves several clinical parameters. An improved metabolic clearance is accomplished in comparison with a CHD schedule [14–17]. Also, previous studies found decreases in blood pressure, left ventricular mass and increased protein intake and survival [15–19]. Another beneficial aspect is the enormous increase in spare time during daytime when dialysis is performed during the night. Whether physical performance actually improves when patients switch from CHD to NHD or when patients on NHD are compared with patients on CHD has been investigated but results are conflicting. In this paper we systematically review the evidence available since the application of NHD, focusing on the research question: is physical performance in hemodialysis patients improved by NHD compared to CHD?

## Materials and methods

### Study protocol

We followed Preferred Reporting Items for Systematic Reviews and Meta-Analyses (PRISMA) guidelines for reporting our data [20] and worked according a pre-documented protocol (Additional file 1).

### Search strategy

A literature search, with help of a specialized librarian, was conducted in multiple databases: MEDLINE (PubMed), Embase, CINAHL, PhycInfo and Web of Science. Databases were searched until 1 January 2018. The following MeSH terms were used: home hemodialysis, renal dialysis, movement, locomotion, motor activity, exercise, physical fitness, physical endurance, physical therapy modalities, physical exertion, recreation, gait, muscle strength, resistance training, sports, early ambulation, exercise movement techniques, exercise therapy. All MeSH terms were used in combination with free-text terms. A draft of the search strategy for Embase is available as supplementary data (Additional file 2).

### Eligibility criteria

Studies were suitable when containing the following terms: 1) nocturnal and/or long hemodialysis, 2) physical performance and/or physical activity and 3) adults (≥18 years). Exclusion criteria were studies regarding frequent, but short (daily) dialysis sessions and studies regarding physical performance or activity in dialysis patients not treated with NHD or long hemodialysis. Studies presenting original study data were included, no further methodological criteria were established because of pre-expected limited search results. No restrictions regarding language, publication year or length of follow-up were made.

### Study and data collection

At first, eligibility was assessed independently by two reviewers (M.D., B.J.) starting with screening of titles and abstracts. The reviewers were not blinded for author or journal. After the screening the reviewers discussed their differences and a consensus was reached. Next, full-text of all articles were searched using a data-extraction form collecting information on study characteristics, demographics, clinical parameters and relevant outcomes. Also, the two reviewers hand-searched bibliographies of relevant publications.

### Risk of bias and quality assessment

The two reviewers independently performed a quality assessment, using the Newcastle-Ottawa scale (NOS) for cohort studies [21]. This quality tool was chosen because knowledge of the literature learned that most studies were expected to be observational cohort studies. The scale of this quality tool consists of 3 components on which a maximum of 9 points can be given on the items patient selection (max. of 4 points), comparability (max. 2 points) and outcome (max. 3 points).

## Results

### Search results

**Fig. 1:**
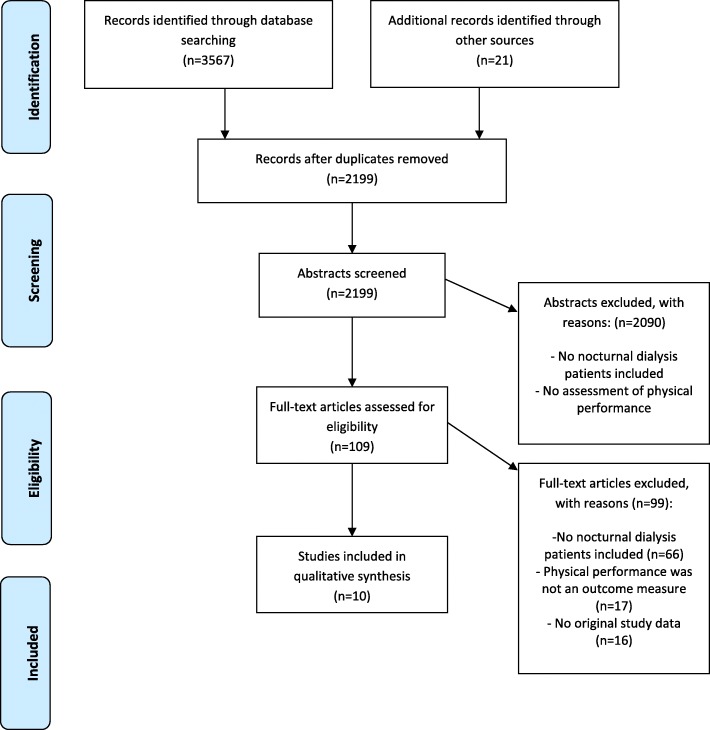
PRISMA flowchart of the selection and screening process

Through database and hand-searching 3588 articles were found of which 2199 articles remained after removing duplicates. Subsequently, 2090 articles were excluded because the abstract revealed that the study did not meet the inclusion criteria. Full-text was assessed of 109 articles, of which 99 articles were excluded based on several reasons, such as no inclusion of NHD patients, no assessment of physical performance or lean mass or function as an outcome measure or no original study data. Ten studies remained for quality syntheses. Figure 1 depicts the screening process.

### Study characteristics

A total of 526 patients were included in ten selected studies. Two studies were randomized controlled trials (RCT) [22, 23]. Two studies were prospective cohort studies in which the change in outcome from CHD to NHD was evaluated [24, 25]. Two studies investigated data cross-sectional, thus comparing patients treated with NHD to patients on CHD [26] or with patients on peritoneal dialysis [27]. Three studies combined the assessment of change in outcome from CHD to NHD, and also used a control group, being CHD patients in 2 studies [14, 28] or healthy controls [29]. One study investigated change in patients already on NHD to a control group of CHD patients [30]. Regarding the treatment with NHD: in 2 studies patients were subjected to NHD during 3 nights a week, 8 h per session [14, 30], 7 studies investigated a more intensive frequency of NHD, thus more than 3 sessions per week of 6–8 h per session [22–24, 26, 28, 29]. One study did not mention the NHD frequency or hours of the dialysis treatment [27]. The follow-up duration ranged from 2 to 60 months. All studies included more males (range 55 to 100%) than females and the study population were between mean 41 to 52 years of age. Study characteristics are described in Tables 1 and 2.

**Table 1:** Characteristics and results of the included studies

Author, year	Country	No. of nocturnal HD (NHD) pts	Frequency of NHD treatment	Study design, control group	Follow-up duration, months	Male %	Age, years	Assessment and outcome	Effects NHD
Performance capacity tests									
Hall, 2012 [22]	USA, Canada	45	6 times/wk., ≥6 h	RCT, CHD controls	12	64	52 ± 14	Short physical performance battery (SPPB), scale 1 to 12	Change SPPB score after 12 mo NHD: 8.1 ± 2.8 to 7.8 ± 3.4. Adjusted mean change in 12 mo NHD: − 0.92 ± 0.44 vs control group: − 0.41 ± 0.43, *p* = 0.41.
Chan, 2007 [29]	Canada	13	5–6 times/wk., 6–8 h	Prospective cohort, healthy controls	6	85	41 ± 3	Exercise duration in seconds and capacity expressed as % of the predicted VO_2peak_	Change in exercise duration after 3–6 mo NHD: 617 ± 50 vs 682 ± 55, *p* = 0.03, VO_2peak_: 66 ± 8 vs 75 ± 6, *p* = 0.05. Healthy controls at baseline: exc. Duration: 722 ± 53, VO_2peak_: 90 ± 4.
Eps, 2010 [24]	Australia	63	3–5 times/wk., 6–10 h	Prospective cohort, no controls	12	79	52 ± 13	6-min walk test (6MWT) in meters	Change 6MWT after 6 mo NHD: 513 m vs 536.5 m, *p* = 0.007.
Lean mass assessments									
Kayson, 2012 [23]	USA, Canada	45	6 times/wk., ≥6 h	RCT, CHD controls	12	64	52 ± 14	Lean body mass (LBM) in kilograms by bioelectrical impedance analysis (BIA)	Mean change LBM after 12 mo NHD: 47.4 ± 12.5 to 48.2 ± 12.0. Adjusted mean change in 12 mo NHD: − 0.49 ± 0.63 vs control group: − 0.04 ± 0.61, *p* = 0.61.
Torigoe, 2016 [30]	Japan	8	3 times/wk., 8 h	Prospective cohort, CHD controls	2	100	45 ± 3	Skeletal muscle mass (SMM), lean body mass (LBM) in kilograms by BIA	Change in SMM after 2 mo NHD: 17 g increase. LBM: 20 g increase after 2 mo NHD, ‘significant’ (no *p*-value).
Ipema, 2014 [28]	Netherlands	11	4–6 times/wk., 8 h	Prospective cohort, CHD controls	12	55	41 (36–51)	Fat-free mass (FFM) in kilograms by Dual-energy X-ray (DXA)	Change FFM after 12 mo NHD: 52.3 ± 8.3 to 50.9 ± 8.50, *p* = 0.095. Change control group: 53.5 ± 8.1 to 52.4 ± 7.6.
Pellicano, 2010 [26]	Australia	28	3–5 times/wk., 8 h	Cross-sectional, matched, CHD controls	–	86	49 ± 11	SMM in kilograms by DXA	Change in SMM in NHD: 26.3 ± 4.16 vs control group: 25.6 ± 5.61, *p* = 0.65.

**Table 2:** Characteristics and results of the studies assessing physical performance with QOL

Author, year	Country	No. of NHD pts	Frequency of NHD treatment	Study design, control group	Follow-up duration, months	Male %	Age, years^a^	Assessment and outcome	Effects NHD
Self-reported measurements									
Hall, 2012 [22]	USA, Canada	45	6 times/wk., ≥6 h	RCT, CHD controls	12	64	52 ± 14	Physical health composite (PHC) by Short form-36 (SF-36), physical functional subscale (PF)	Change PHC after 12 mo NHD: 2.7 ± 1.4 vs control group: 2.1 ± 1.5, *p* = 0.75, Change PF after 12 mo NHD: − 3.1 ± 3.5 vs control group: 1.1 ± 3.6, *p* = 0.40.
Ok, 2014 [14]	Turkey	247	3 times/wk., 7–8 h	Prospective, non-randomized case-control, CHD controls	12	68	45 ± 14	Physical functioning by SF-36	Described: all dimensions were unchanged in the NHD group. Change in control group: vitality score decreased, 68.7 ± 24.3 to 64.4 ± 25.2, *p* = 0.01.
Eps, 2010 [24]	Australia	63	3–5 times/wk., 6–10 h	Prospective cohort, no controls	6–12	79	52 ± 13	Physical functioning by Kidney disease QoL (KD-QoL)	Change in PF after 6–12 mo NHD: 60 to 75, *p* = 0.003.
Lockridge, 2004 [25]	Canada	40	5–6 times/wk., 6–10 h	Prospective, longitudinal, no controls	60	65	50 (23–81)	Physical composite score (PCS) by SF-36	Change in overall mean PCS score after 5 yrs. NHD: 35.23 to 44.94, *p* = 0.007.
Fong, 2007 [27]	Canada	26	–	Cross-sectional, PD controls	–	67	4912	Physical component summary by KD-QoL	PCS in NHD: 55 ± 2.3 vs control group (PD): 52.3 ± 1.8, *p* = 0.35.

^a^Age is given as mean ± SD or as median and IQR

### Risk of bias assessment

**Fig. 2:**
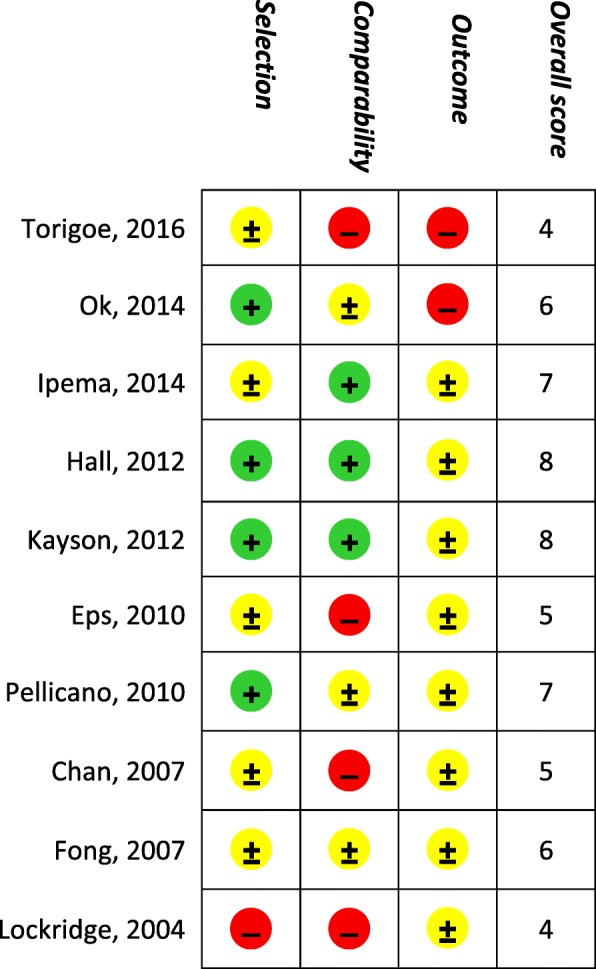
Risk of bias assessment of the 10 included studies, according to the 3 components of the Newcastle-Ottawa scale

Results of a risk of bias assessment are given in Fig. 2. Several studies had a small sample size (15 subjects or less) [28–30]. Except for one study [27], all studies described NHD frequency and weekly hours of treatment. Seven studies describe their in- and exclusion criteria [14, 22, 23, 25, 26, 28], 4 studies described limited or no information regarding in- and exclusion criteria [24, 27, 29, 30]. Six studies reported limited or no information regarding the recruitment process [24, 26–30]. Assessment of outcome was reported clearly in most studies, e.g. frequency of the assessment, which type of tool was used for the assessment and performed before or after dialysis. One study described limited information regarding outcome measurements [30]. Three studies provided insufficient information on subjects’ follow-up, such as which patients completed follow-up and reasons why subjects did not completed the study [25, 29, 30].

### Performance capacity tests

Three studies assessed physical performance by capacity tests. One study used the short-physical performance battery (SPPB) and did not find an improvement after 1 year of treatment [22]. One study used the 6-min walk test (6MWT) and found an improvement after 6 months of NHD [24]. The study assessing physical performance with exercise spirometry (VO_2peak_) and exercise duration found a significant improvement after 3–6 months of NHD [29].

### Lean mass assessments

Four studies assessed lean mass by different methodology and described lean mass with different terminology. One study assessed lean mass as dual-energy X-ray absorptiometry (DXA) derived fat free mass [28] and one study as DXA derived skeletal muscle mass [26]. Both reported no change in lean mass. One study assessed lean mass as bioelectrical impedance analysis (BIA) derived lean body mass and reported no change after 1 year [23]. One study assessed BIA derived lean mass and described an increased lean body mass and skeletal muscle mass [30].

### Self-reported physical performance

Five studies investigated physical performance as part of a QoL questionnaire. Two of these used the Kidney Disease Quality of Life (KD-QoL) questionnaire [24, 27], of which one found a significant improvement of the physical component score after 6 to 12 months of NHD treatment [24]. Three studies used the Short-Form 36-item health survey (SF-36) to assess physical performance [14, 22, 25]. One of these found that the physical component scores after 5 years of NHD improved significantly [25], the other two studies, with a follow-up of 1 year, found no changes in physical component scores [14, 22, 27].

## Discussion

In this systematic review, we summarize the effect of NHD compared to CHD on physical performance. About half of the included studies did not find an effect of NHD, including the RCTs, whereas some studies did find slight effects on physical activity endurance, muscle mass and the self-reported physical component score of QoL.

Three studies assessed physical performance with physical capacity tests [22, 24, 29]. Two of these studies used a 6MWT and a bicycle test to establish exercise duration [24, 29]. In these studies an improvement was found in walking distance after 12 months of NHD and exercise duration after 6 months of NHD [24, 29]. However, the Frequent Hemodialysis Network trial assessed physical performance in an RCT using the SPPB and found no difference after 12 months of NHD in comparison with patients on maintenance hemodialysis [22]. The authors mentioned difficulties when recruiting NHD patients, which resulted in a smaller sample size and possible lack of power. In our opinion, this result might be more affected by the choice of assessment. Previous studies do have shown that improvements in physical performance can be detected by the SPPB, but these changes were found in older, less healthy patients [31, 32]. Patients who start a nocturnal (home) dialysis treatment are general quite fit and in a stable medical condition. One could question the sensitivity of SPPB as outcome parameter in NHD patients, considering a possible ceiling effect in the SPPB [33]. If we would exclude the study with SPPB as outcome parameter, then the performance capacity tests show a slight improvement with NHD although this conclusion is based on only two studies. Combining SPPB with more challenging tests, such as a 6MWT, a shuttle-run test and/or a bicycle test, would create a test-battery with different activity levels, preventing a ceiling effect and increasing responsiveness.

Four studies assessed lean mass with DXA or BIA [23, 26, 28, 30]. Although assessment of body composition might not be a direct measurement of physical performance, muscle mass is an important supporting aspect of physical performance. For example, resistance exercise does contribute to an increase in lean mass [12, 13, 34]. Of the 4 included studies investigating lean mass, 2 studies found no difference after 1 year of NHD in comparison with CHD [23, 28] and 2 studies did find an improvement after NHD compared to CHD [26, 30]. Unfortunately, information on physical activity or training patterns is not available in these studies. Combining data on lean mass, physical activity and training would have given optimal insight in a patient’s ability to increase his or her lean mass over time.

Five studies investigated physical performance with a QoL questionnaire [14, 22, 24, 25, 27]. Only two studies found a significant improvement of the physical component score in NHD patients [24, 25]. Again, the question might be if the QoL questionnaire is sensitive enough to detect small changes over time in physical performance in this population. The QoL questionnaires does include questions regarding daily activities, but most questions focus on relatively easy daily activities, such as the capability of walking a stair or walking 100 m. Components of exercise, such as hiking, cycling or performing sports and the frequency of these activities, are not or very limited taken into account in the QoL questionnaires. Again, patients who are joining a nocturnal program, might be relatively fit and questions of a QoL questionnaire might not discriminate enough to detect the differences in this population.

A major limitation of the investigations published on this subject are the different assessments that are used across all studies to assess physical performance and/or supporting muscle mass. Because studies use different methods and vary between the use of performance tests versus self-reported tests, it is difficult to make a valid comparison between studies. Establishing a consensus about a gold standard for a test or test-battery to assess physical performance would improve the quality of studies in the future and leads to better comparability of studies.

Our results are influenced by insufficient description of methodological information of some included studies. We assessed the risk of bias based on the described information from each paper, but some studies did not describe all components of their work. Certain baseline characteristics, the recruitment process or follow-up of subjects during the study, were not or insufficiently described. Therefore, the risk of possible bias, such as selection bias, is present which prohibits a good estimation of the quality of some studies.

## Conclusion

In conclusion, the limited amount of studies and the limited methodological quality of the studies prohibits a firm assessment of improved physical performance by NHD compared to CHD. As current literature emphasizes the importance of physical performance on clinical outcomes, it is essential to conduct more and high quality research. We recommend to combine physical capacity tests, measurement of body composition and self-reported measurements in order to construct a valid comprehensive assessment of physical performance. In addition, the range of scoring should be broad enough in order to increase discriminative power and responsiveness of the test. We advocate to develop a gold standard for assessing physical performance in ESRD patients.

## Additional files

Additional file 1: Protocol (DOCX 18 kb)

Additional file 2: Example of the search strategy for Embase (DOCX 12 kb)

## Data Availability

Not applicable.

## Abbreviations

6MWT: 6-min walk test
BIA: bioelectrical impedance analysis
CHD: conventional hemodialysis
DXA: dual-energy X-ray absorptiometry
ESRD: end-stage renal disease
HD: hemodialysis
KDQOL-SF: kidney disease quality of life-short form
NHD: nocturnal hemodialysis
NOS: Newcastle-Ottawa scale
PRISMA: Preferred Reporting Items for Systematic Reviews and Meta-Analyses
QoL: quality of life
RCT: randomized controlled trial
SF-36: Short-Form 36-item health survey
SPPB: short physical performance battery
