# Supplementary material for: Physical performance in patients treated with nocturnal hemodialysis - a systematic review of the evidence
Source: BMC Nephrol. 2019 Aug 14;20:317. doi: 10.1186/s12882-019-1518-4 (PMC6694635; doi:10.1186/s12882-019-1518-4)
Supplement: Supplementary file 1 — Protocol (DOCX 18 kb) [file 12882_2019_1518_MOESM1_ESM.docx]

**Additional file 1. Protocol.**

**A Systematic Review regarding the effect of nocturnal hemodialysis on physical performance.**

M. Dam, PJM Weijs, FJ van Ittersum, B.C. van Jaarsveld

**Aim**

The aim of this study is to compare patients on conventional hemodialysis and nocturnal hemodialysis regarding physical performance status.

**P** Nocturnal hemodialysis patients

**I** Physical performance interventions or measuring of physical performance status

**C** Patients receiving conventional hemodialysis

**O** Physical performance, physical activity

**In- and exclusion criteria**

**Inclusion:**

Included studies must contain the following factors: nocturnal hemodialysis and physical performance and/or physical activity.

The following terms will be included in the search strategy:

- Nocturnal hemodialysis / haemodialysis
- Night hemodialysis / haemodialysis
- Frequent* hemodialysis / haemodialysis
- Home* hemodialysis / haemodialysis
- Long* hemodialysis / haemodialysis
- Intensive* hemodialysis / haemodialysis
- Physical performance
- Physical activity
- Physical function
- Performance status
- Exercise
- Handgrip strength
- Strength
- Muscle strength
- SPPB / Short Physical Performance Battery
- 6 minute walk test

**These will also be added as additional filters because often these terms are used to describe or include nocturnal hemodialysis.*

*Study types:* Randomized controlled trials, controlled trials, prospective or retrospective cohort studies, observational studies with a control group, case-control studies.

No restriction regarding publication year will be made.

**Exclusion:**

- Studies regarding conventional hemodialysis patients and physical performance or activity who do not plan to switch to nocturnal hemodialysis will be excluded.
- Studies regarding frequent, but short (daily) dialysis session (e.g. 2 hours a day).

**Search strategy**

(1) We will investigate at least the following electronic databases:

- MEDLINE (PUBMED)
- EMBASE
- The Cochrane library

(2) Additional search strategies will include:

- Reference list searching
- Checking Trial Registrations

MESH terms will be used in combination with free-text terms. We will approach a specialized librarian to conduct a comprehensive database search and discuss appropriate databases.

**Study selection and screening**

Two authors (MD, BvJ) will independently screen the potential relevant articles against the inclusion- and exclusion criteria, without blinding the authors. In case of insufficient information based on title or abstract the full-text article will be inspected.

When articles are found eligible to include based on title and abstract, full-text papers will be assessed.

Any uncertainty or disagreements between both authors during title/abstract screening or full-test screening will be solved by a discussion with both researchers and an third researcher (FvI).

All eligible references will be saved in Endnote. Results of the screening will be recorded in a PRISMA-flowchart.

**Quality assessment**

Quality assessment of the full-text papers will be performed using the validated Newcastle-Ottawa scale. A maximum score for this scale is 9 points.

**Reporting**

The systematic review will be reported according to the PRISMA statement.
