# Supplementary material for: Physical performance in patients treated with nocturnal hemodialysis - a systematic review of the evidence
Source: BMC Nephrol. 2019 Aug 14;20:317. doi: 10.1186/s12882-019-1518-4 (PMC6694635; doi:10.1186/s12882-019-1518-4)
Supplement: Supplementary file 2 — Example of the search strategy for Embase (DOCX 12 kb) [file 12882_2019_1518_MOESM2_ESM.docx]

**Additional file 2.**

**Example of the search strategy for Embase**

'home dialysis'/exp OR ((nocturnal* OR night* OR home OR intensive* OR ‘thrice weekly’ OR ‘three times weekly’):ab,ti) AND 'hemodialysis'/de OR hemodialy*:ab,ti OR haemodialy*:ab,ti OR ‘renal dialy*’:ab,ti

'kinesiology'/exp OR 'mobilization'/exp OR 'occupational therapy'/exp OR 'walking difficulty'/exp OR 'accelerometry'/exp OR 'movement (physiology)'/de OR 'limb movement'/exp OR 'walking'/exp OR 'patient mobility'/exp OR 'physical mobility'/exp OR 'voluntary movement'/exp OR 'motor activity'/de OR 'exercise'/exp OR 'physical activity, capacity and performance'/de OR 'endurance'/exp OR 'physical activity'/exp OR 'physical capacity'/exp OR 'physical performance'/exp OR 'training'/exp OR 'fitness'/exp OR 'physiotherapy'/exp OR 'recreation'/de OR 'dancing'/exp OR 'muscle strength'/exp OR 'sport'/exp OR 'kinesiotherapy'/exp

OR

(‘Motor Activit*’ OR ‘Physical Activit*’ OR ‘Locomotor Activit*’ OR Exercis* OR training OR stretching OR ‘Physical Condition*’ OR ‘Physical fitness’ OR ‘Physical endurance’ OR ‘movement therap*’ OR plyometr* OR ‘Stretch-Shortening’ OR ‘Weight-Lift*’ OR ‘Weight-Bearing’ OR running OR jogging OR walk* OR bicycle OR cycle OR bicycling OR cycling OR rowing OR swim* OR ambulation OR mobil* OR pilates OR yoga OR 6mwt OR fitness* OR enduranc* OR capacity OR ‘muscle mass*’ OR acceleromet* OR pedomet* OR mobility OR ambulat* OR gait OR speed OR ‘functional status’ OR functioning OR sppb OR ‘physical performanc*’ OR aerobic* OR strength* OR ‘muscle power’ OR ‘sit to stand test’ OR ‘free weight*’ OR ‘machine weight*’ OR ‘resistance band*’ OR kinesio* OR exerti* OR ‘occupational therap*’ OR running OR gymnastic*):ab,ti
